# Progesterone Upregulates Gene Expression in Normal Human Thyroid Follicular Cells

**DOI:** 10.1155/2015/864852

**Published:** 2015-05-21

**Authors:** Ana Paula Santin Bertoni, Ilma Simoni Brum, Ana Caroline Hillebrand, Tania Weber Furlanetto

**Affiliations:** ^1^Programa de Pós Graduação em Medicina: Ciências Médicas, Universidade Federal do Rio Grande do Sul, Rua Ramiro Barcelos 2350/700, 90035-903 Porto Alegre, RS, Brazil; ^2^Programa de Pós Graduação em Ciências Biológicas: Fisiologia, Instituto de Ciências Básicas da Saúde, Universidade Federal do Rio Grande do Sul, 90050-170 Porto Alegre, RS, Brazil

## Abstract

Thyroid cancer and thyroid nodules are more prevalent in women than men, so female sex hormones may have an etiological role in these conditions. There are no data about direct effects of progesterone on thyroid cells, so the aim of the present study was to evaluate progesterone effects in the sodium-iodide symporter *NIS*, thyroglobulin *TG*, thyroperoxidase *TPO*, and *KI-67* genes expression, in normal thyroid follicular cells, derived from human tissue. *NIS*, *TG*, *TPO*, and *KI-67* mRNA expression increased significantly after TSH 20 *μ*UI/mL, respectively: 2.08 times, *P* < 0.0001; 2.39 times, *P* = 0.01; 1.58 times, *P* = 0.0003; and 1.87 times, *P* < 0.0001. In thyroid cells treated with 20 *μ*UI/mL TSH plus 10 nM progesterone, RNA expression of *NIS*, *TG*, and *KI-67* genes increased, respectively: 1.78 times, *P* < 0.0001; 1.75 times, *P* = 0.037; and 1.95 times, *P* < 0.0001, and *TPO* mRNA expression also increased, though not significantly (1.77 times, *P* = 0.069). These effects were abolished by mifepristone, an antagonist of progesterone receptor, suggesting that genes involved in thyroid cell function and proliferation are upregulated by progesterone. This work provides evidence that progesterone has a direct effect on thyroid cells, upregulating genes involved in thyroid function and growth.

## 1. Introduction

Thyroid nodules and thyroid cancer are more common in women [1, 2]. The incidence rate of thyroid cancer (TC) is increasing, which could be due to better surveillance techniques [3]. Nevertheless, other factors are probably involved because the incidence rates of tumors of all sizes increased [4]. As the increase in incidence of TC appears to be higher in women, sex hormones might have an etiological role [5]. Reproductive factors, as history of uterine fibroids, greater number of live births, greater number of reproductive years, and greater estimated number of ovulatory cycles, were associated with an increased risk of TC [6]. Also, during pregnancy, thyroid nodules were shown to increase in number and size [7, 8], and TC has been shown to affect one in a thousand pregnant women, being the second most common type of cancer in pregnancy [9].

Several studies have shown thyroid cells growth in response to estrogen [10–13], as reviewed recently [14], and some data suggest a decrease in differentiated function by this hormone in thyroid cells [11, 15].

There are no studies of progesterone effects on thyroid cells. Progesterone might have a protective effect on the thyroid because the use of oral contraceptives and menopausal hormone therapy, with the combination of estrogen and progesterone, were not associated with thyroid cancer risk [6], but irregular menstrual bleeding, usually due to anovulatory cycles, has been associated with it [16].

Although progesterone could exert its effects through genomic or nongenomic mechanisms [17, 18], its primary action is to regulate the expression of target genes through binding directly to its cognate sequence in the genes, called progesterone responsive elements [19]. As PR have been variably described in thyroid follicular cells [20–23], the aim of the present study was to evaluate the effects of progesterone on gene expression in a model of normal human thyroid cells in primary culture.

## 2. Methods 

### 2.1. Reagents

All reagents were purchased from Sigma-Aldrich unless otherwise specified.

### 2.2. Thyroid Tissue Acquisition

Tissue samples were obtained from patients submitted to thyroidectomy as part of treatment for differentiated TC. After evaluation by two pathologists, normal tissue that would have been discharged was kept in Hank's solution at about 4°C until processing. The project was submitted to, and approved by, the Research Ethics Committee of the Hospital de Clínicas de Porto Alegre, Porto Alegre, RS, Brazil (CEP: 09-454), which waived a written informed consent. In accordance with the Resolution HCPA 02/97, based on the Resolution CNS 196/96 of the National Health Council, Brazil, and the Guideline 9 of the International Ethical Guidelines for Biomedical Research Involving Human Subjects (CIOMS, WHO, Geneva, 1993), there was no need to obtain informed consent of the patients, because only after the surgical procedure the researchers would know if a tissue sample would be available, they would not know the identity nor had access to the files of the patients, and the tissue samples included would have been discharged by the pathologists. The authors signed the “Statement of commitment to use of data,” which states that no data that could identify patients would be used and also that their anonymity would be preserved in any publication of the results.

#### 2.2.1. Primary Culture of Normal Thyroid Cells

Thyroid follicular cells isolation and culture were performed as described previously [24]. Briefly, thyroid tissue was cut in fragments of about 1 mm^3^ and digested by 3 mg/mL type I collagenase (GIBCO, Grand Island, NY, USA). The suspension of cells was sequentially filtered through nylon meshes (250, 150, and 60 *μ*m pore size) and the filtered fraction, containing epithelial thyroid cells, was resuspended and seeded in a 35 mm Petri dish at a density of 1 × 10^6^ cells/cm^2^. Thyroid cells were cultured initially in Ham's F-12 Coon's modification medium supplemented with 10% fetal bovine serum, 10 *μ*g/mL insulin, 5 *μ*g/mL transferrin, 1 mU/mL TSH, and 100 U/mL kanamycin at 37°C with 5% CO_2_; after two changes, the concentration of serum was decreased to 5% (3H medium).

#### 2.2.2. Treatments

Five or 7 days later, cells were deprived of TSH for 48 hours (3H medium minus TSH: 2H medium); subsequently, they were treated with 2H medium and progesterone, mifepristone, or vehicle ethanol, as follows: Group 1: 2H medium; Group 2: 2H medium + 20 *μ*U/mL TSH; Group 3: 2H medium + 20 *μ*U/mL TSH + 10 nM progesterone, and Group 4: 2H medium + 20 *μ*U/mL TSH + 10 nM progesterone + 100 nM mifepristone, for 4 h or 48 h, before total RNA isolation. Ethanol was added so the final concentration was 0.1% for all groups.

#### 2.2.3. Gene Expression Analysis

Total RNA was isolated using the Trizol reagent (Invitrogen Life Technologies, Carlsbad, CA, USA). Concentration and purity were assessed by the Nanodrop ND-1000 spectrophotometer (Nanodrop Technologies, Rockland, DE, USA) and stored at −80°C. 1 *μ*g total RNA was transcribed into cDNA by Superscript III Reverse Transcriptase (Invitrogen Life Technologies) and stored at −20°C.

Expression of* KI-67*, thyroglobulin* TG*, thyroperoxidase* TPO*, and sodium-iodide symporter* NIS* genes was assessed by quantitative reverse transcriptase polymerase chain reaction (RT-qPCR) on Applied Biosystems StepOne Real-Time PCR System using Kit Platinum SYBR Green qPCR SuperMix-UDG (Invitrogen Life Technologies). *β*-actin was used as reference gene [24]. The primers used for the specific amplification of* TG*,* TPO*, and* NIS* were described previously [25]. The sequences of the primers for progesterone receptor were 5′-ACCCGCCCTATCTCAACTACC-3′ and 5′-AGGACACCATAATGACAGCCT-3′. Annealing temperature of 60°C was used for amplification; dissociation curves were performed by running a gradient of 60–95°C to confirm the specificity of the PCR amplification. All samples were amplified in triplicate, and cDNA standard curves were constructed using the threshold cycles with five successive 10-fold dilution points of a pool of cDNA samples.

### 2.3. Statistical Analysis

RT-qPCR experiments were performed in duplicate and repeated in three (*NIS* gene) or four (*KI-67*,* TG*, and* TPO* genes) independent experiments, with cells isolated from different patients. mRNA values were normalized by *β*-actin, before statistical analysis. Differences in the means of the treatment groups were evaluated with the generalized estimating equation (GEE). After GEE, post hoc Bonferroni test was used to identify which means were different. All statistical analyses were performed with SPSS software, version 18.0 (SPSS, Chicago, IL), and considered significant when *P* was less than 0.05.

## 3. Results

### 3.1. Thyroid Cells in Primary Cultures Were Responsive to Stimulation by TSH

**Figure 1:**
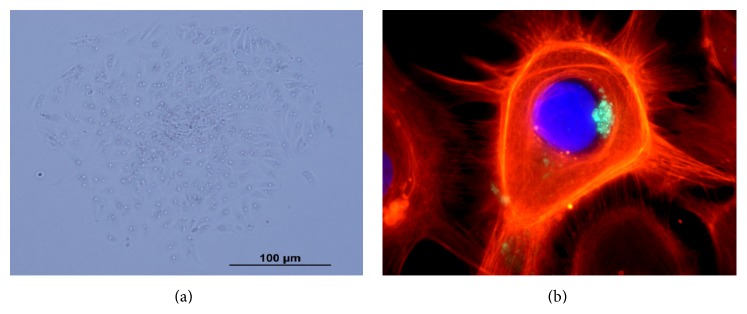
Normal human thyroid follicular cells in primary culture. (a) Thyrocytes in monolayers showing characteristic morphology by phase contrast microscopy and (b) immunocytochemistry of actin with phalloidin-fluorescein isothiocyanate (red), nuclei stained with DAPI (blue), and thyroperoxidase protein (green).

Thyroid follicular cells viability and differentiation were assessed by characteristic morphology and by staining TPO protein in immunocytochemistry. As showed in Figure 1(a), the thyroid follicular cells isolated in our model present a cuboid shape, with the presence of many vacuoles around the nucleus as observed in previous studies of primary culture of follicular cells thyroid [26]. Also, as other epithelioid cells, these cells grow as islands and form a monolayer with extensive cell-to-cell contact. In Figure 1(b), TPO protein staining, characteristic of thyroid follicular cells, is shown. The specific thyroid genes expression (*TG*,* TPO*, and* NIS*) was also positive in our primary culture of thyroid follicular cells.

**Figure 2:**
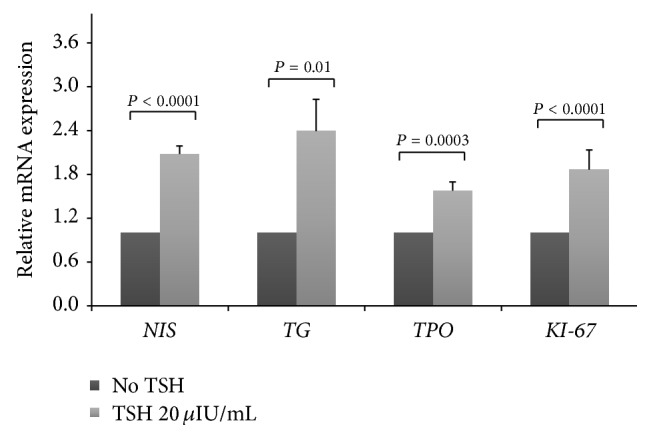
TSH increased mean mRNA expression of human normal thyroid follicular cells genes in primary culture. The sodium-iodide symporter* NIS* gene was evaluated after 4 h, and the thyroglobulin* TG*, thyroperoxidase* TPO*, and* KI-67* genes were evaluated after 48 h. Data were obtained by RT-qPCR and normalized with *β*-actin gene expression and are shown as the mean ratio of TSH/no TSH ± standard deviation, considering no TSH = 1. The experiments were performed in duplicate and repeated in three independent cultures for* NIS* and four independent cultures for* TG*,* TPO*, and* KI-67*.

TSH increased significantly the* NIS* gene expression (2.08 times) after 4 h; it also increased significantly the* TG* (2.39 times),* TPO* (1.58 times), and* KI-67* (1.87 times) genes expression, after 48 h, as shown in Figure 2.

### 3.2. Progesterone Upregulated* NIS*,* TG*,* TPO*, and* KI-67* mRNA Expression in Normal Human Thyroid Follicular Cells

**Figure 3:**
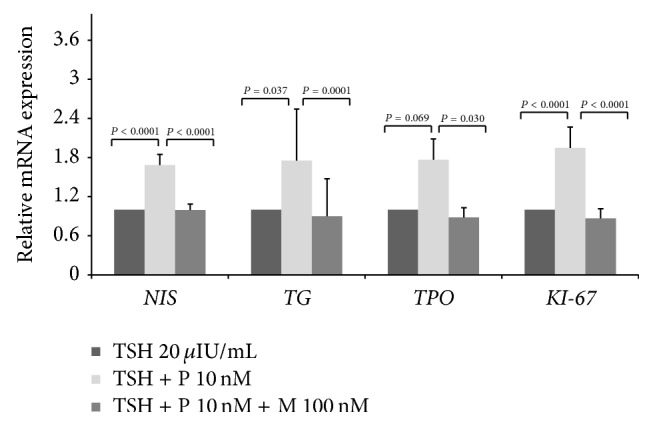
Progesterone (P) increased mean mRNA expression of human normal thyroid follicular cells genes in primary culture. The sodium-iodide symporter* NIS* gene was evaluated after 4 h, and the thyroglobulin* TG*, thyroperoxidase* TPO*, and* KI-67* genes were evaluated after 48 h. These effects were abolished by mifepristone (M). Data were obtained by RT-qPCR and normalized with *β*-actin gene expression and are shown as the mean ratio of treatment/TSH ± standard deviation, considering TSH = 1. The experiments were performed in duplicate and repeated in three independent cultures for* NIS* and four independent cultures for* TG*,* TPO*, and* KI-67*.

To evaluate the effect of progesterone treatment on the expression of* NIS*,* TG*,* TPO*, and* KI-67* genes, RT-qPCR was applied. Gene expression of progesterone receptor was present in all thyroid cells cultures used in this study (data not shown). When thyroid cells were treated with 20 *μ*UI/mL TSH plus 10 nM progesterone, the mean expression of* NIS* mRNA after 4 h, and the mean expression of* TG*,* TPO*, and* KI-67* mRNA after 48 h, increased, respectively, by 1.75, 1.77, 1.68, and 1.95 times, when compared to the group treated with TSH (Figure 3).

## 4. Discussion

In the present study, for the first time, an effect of progesterone on thyroid cells was demonstrated. Genes involved in the differentiated protein expression of thyroid cells were upregulated by progesterone, as well as* KI-67*, a marker for growth; these effects were abolished by mifepristone.

Isolated human follicular cells derived from normal thyroid tissue have been studied previously [27, 28], with high functional correspondence with the original follicular cells. In our model, thyroid cells were grown in monolayer and exhibited the expected morphology.* TG*,* TPO*, and* NIS* mRNA were detected even when the cells were deprived of TSH for 48 h, which could be due to other components of the medium and its supplements, such as insulin, or constitutive activation of thyroid cells [29].

It is known that thyroid cell regulation varies in different species, and, sometimes, the cells can have different phenotypes and responses to TSH [29, 30]. In our model, normal human thyroid follicular cells in primary culture were shown to be differentiated and responsive to TSH. The response of gene expression to TSH has been shown to vary with the gene and time after treatment. Our cells responded to TSH, as expected [11, 31–37].

As no direct effect of progesterone on thyroid cells has been described until now, we have no other studies to compare our results with. As progesterone increased the expression of genes involved in growth and differentiated function of thyroid cells, its effect on the thyroid gland could be protective. Recently Sathi et al. observed an increase in free T4 levels in progesterone-treated women, and a tendency towards lower TSH levels during progesterone treatment, in a randomized controlled progesterone trial [38].

Our results suggest that progesterone effects on human thyroid cell occur via a transcriptional level through its nuclear receptor, since a specific antagonist, mifepristone, abolished it. Mifepristone exerts its antiprogesterone effects by binding PR with higher affinity than progesterone [39]. The ability of mifepristone to abolish progesterone effect in thyroid cells suggests that these receptors are functional in these cells, although its concentration in normal thyroid has been described as low or undetectable [22, 23]. In a recent study PR expression was seen in 75.8% of patients with papillary thyroid cancer [40]. In our study, PR gene expression was identified in all cell cultures.

As mifepristone blocks also the action of glucocorticoids and some synthetic progestogens, as medroxyprogesterone, are ligands for the glucocorticoids receptor (GR), the effects described in the present study could have been mediated by these receptors. Nevertheless, the ability of progesterone to bind to GR is disputable [41].

Although the model used in the present study has contributed to the understanding of thyroid cell growth regulation [42], the increase in gene expression does not always result in a proportional increase in functional proteins. Besides, our monolayer model does not reproduce the complex organization of thyrocytes in the follicle, so functional and growth studies are necessary to better understand a possible role of progesterone in thyroid cells.

## 5. Conclusion

This work provides evidences that progesterone has a direct effect on thyroid cells, through its receptor, upregulating genes involved in thyroid function and growth.
